# HPV Vaccination: The Position Paper of the Italian Society of Colposcopy and Cervico-Vaginal Pathology (SICPCV)

**DOI:** 10.3390/vaccines8030354

**Published:** 2020-07-02

**Authors:** Andrea Ciavattini, Luca Giannella, Rosa De Vincenzo, Jacopo Di Giuseppe, Maria Papiccio, Ankica Lukic, Giovanni Delli Carpini, Antonio Perino, Antonio Frega, Francesco Sopracordevole, Maggiorino Barbero, Murat Gultekin

**Affiliations:** 1 Gynecologic Section, Department of Odontostomatologic and Specialized Clinical Sciences, Università Politecnica delle Marche, 60121 Ancona, Italy; luca.giannella@ospedaliriuniti.marche.it (L.G.); Jacopo.digiuseppe@ospedaliriuniti.marche.it (J.D.G.); maria.papiccio@gmail.com (M.P.); Giovanni.DelliCarpini@ospedaliriuniti.marche.it (G.D.C.); 2 Gynecologic Oncology Unit, Fondazione Policlinico Universitario A. Gemelli, IRCCS, Dipartimento Scienze della Salute della Donna, del Bambino e di Sanità Pubblica, 00168 Rome, Italy; rosa.devincenzo@unicatt.it; 3 Dipartimento di Scienze della vita e Sanità Pubblica, Università Cattolica del Sacro Cuore, 00168 Rome, Italy; 4 Department of Surgical and Medical Sciences and Translational Medicine, Sapienza University of Rome, S. Andrea Hospital, 00185 Rome, Italy; ankica.lukic@uniroma1.it (A.L.); a.frega@tin.it (A.F.); 5 Department of Sciences for Health Promotion and Mother-Child Care “G. D’Alessandro”, Polyclinic University Hospital, 90127 Palermo, Italy; antonio.perino@unipa.it; 6 Gynecological Oncology Unit, Centro di Riferimento Oncologico-National Cancer Institute, 33081 Aviano, Italy; fsopracordevole@cro.it; 7 Department of Obstetrics and Gynecology, Asti Community Hospital, 14100 Asti, Italy; barberom@tin.it; 8 Gynecology and Obstetrics, Division of Gynecologic Oncology, Hacettepe University Faculty of Medicine, 06100 Ankara, Turkey; mrtgultekin@yahoo.com

**Keywords:** human papillomavirus, cervical cancer, cervical intraepithelial neoplasia, HPV vaccine, primary prevention

## Abstract

Human papillomavirus (HPV) related cervical cancer represents an issue of public health priority. The World Health Organization recommended the introduction of HPV vaccination in all national public programs. In Europe, vaccines against HPV have been available since 2006. In Italy, vaccination is recommended and has been freely offered to all young girls aged 11 years since 2008. Three prophylactic HPV vaccines are available against high- and low-risk genotypes. The quadrivalent vaccine contains protein antigens for HPV 6, 11, 16, and 18. The bivalent vaccine includes antigens for HPV 16 and 18. The nonavalent vaccine was introduced in 2014, and it targets HPV types 6, 11, 16, 18, 31, 33, 45, 52, and 58. Clinical trials demonstrated the effectiveness of the three vaccines in healthy young women. Likewise, all vaccines showed an excellent safety profile. The bivalent vaccine provides two doses in subjects aged between 9 and 14 years and three doses in subjects over 14 years of age. The quadrivalent vaccine provides two doses in individuals from 9 to 13 years and three doses in individuals aged 14 years and over. The nonavalent vaccine schedule provides two doses in individuals from 9 to 14 years of age and three doses in individuals aged 15 years and over at the time of the first administration. Preliminary results suggest that the HPV vaccine is effective in the prevention of cervical squamous intraepithelial lesions even after local treatment. Given these outcomes, in general, it is imperative to expand the vaccinated target population. Some interventions to improve the HPV vaccine’s uptake include patient reminders, physicians-focused interventions, school-based vaccinations programs, and social marketing strategies. The Italian Society of Colposcopy and Cervico-Vaginal Pathology (SICPCV) is committed to supporting vaccination programs for children and adolescents with a catch-up program for young adults. The SICPCV also helps clinical and information initiatives in developing countries to decrease the incidence of cervico-vaginal and vulvar pathology.

## 1. HPV and Cervical Cancer

Human papillomavirus (HPV) related cervical cancer represents an issue of public health priority. Five hundred twenty-eight thousand cases of cervical cancer and 266,000 deaths are reported each year worldwide (more than 85% in developing countries). In Europe, HPV-related diseases include 35,000 cervical cancers and 10,000 vulvar and vaginal cancers per year [1]. According to the Aiom-Airtum 2017–2018 data in Italy, cervical cancer has an incidence of 2400 new cases every year [2]. Furthermore, almost 5000 new cases/year of tumors involving the uterine cervix, anus, vagina, vulva, penis, oral cavity, pharynx, and larynx are attributed to chronic infections of oncogenic genotypes of HPV [2,3,4].

The International Agency for Cancer Research (IARC) has identified 12 HPV genotypes as causal agents of cervical cancer (genotypes 16, 18, 31, 33, 35, 39, 45, 51, 52, 56, 58, and 59) and another eight genotypes that could have a potential role in cervical cancer development (genotypes 26, 53, 66, 67, 68, 70, 73, and 82) [5]. In a meta-analysis including 61 countries worldwide, HPV types 16 and 18 caused 70% of cervical cancers and 52% of high-grade lesions [6].

In the Italian population, the most common HPV genotype in cervical cancer is HPV 16 [7]. HPV16 is the most common HPV genotype in other cancer types in Italy, including vulvar cancer and oropharyngeal cancer [8,9]. The prevalence of HPV 16 in the healthy Italian population is around 5% (2–10%), while HPV 18 is present in about 1% (0–6%) [10]. An interesting Italian study showed that the seroprevalence of HPV 6-11-16-18 in healthy girls aged 11–18 years and over 18 was 54.1% and 8.2%, respectively [11]. When cytological or histological lesions are present, the prevalence of these two HPV types increases. HPV 16 positivity is 35% in low-grade cervical intraepithelial neoplasia (CIN) or CIN1, 64% in more severe dysplasia (CIN 2/3), and 68% in cases of invasive cervical carcinoma. HPV 18 positivity is 6% in CIN 1, 7% in CIN 2/3, and 11% in invasive cervical carcinoma. There are no differences in the HPV type distribution between different geographical areas [10,12].

Knowledge of HPV genotype distribution in invasive cervical cancer has been pivotal in developing prophylactic vaccines. The World Health Organization (WHO) recognized the importance of HPV-related disease as a global public health problem. It recommended the introduction of HPV vaccination in all national public programs [13,14]. In Europe, vaccines against HPV have been available since 2006. In Italy, vaccination is recommended and has been freely offered to all young girls aged 11 years since 2008.

Finally, it is useful to emphasize that HPV vaccination is effective for the prevention of vulvar intraepithelial neoplasia (VIN), vaginal intraepithelial neoplasia (VaIN), anal intraepithelial neoplasia (AIN), and their respective cancers [14].

## 2. Effectiveness and Safety of HPV Vaccines

Three prophylactic HPV vaccines are available, directed against high- and low-risk genotypes. The quadrivalent vaccine (4vHPV vaccine) was first introduced in 2006 and contains protein antigens for HPV 6, 11, 16, and 18. The bivalent vaccine (2vHPV vaccine) was introduced in 2007 and contains protein antigens for HPV 16 and 18. The second-generation nonavalent vaccine (9vHPV vaccine) was introduced in 2014, and it targets antigens for HPV types 6, 11, 16, 18, 31, 33, 45, 52, and 58. The three registered vaccines offer comparable immunogenicity and effectiveness for cervical cancer prevention due to the HPV types they cover [13]. Moreover, they have excellent safety profiles [13].

Clinic trials have mainly demonstrated the effectiveness of the three vaccines in healthy young women. The FUTURE I (NCT00092521) study was conducted on women aged 16–24 years. This study randomized 5455 women to receive three doses of the HPV vaccine versus a placebo with a follow-up period of three years. It evaluated the development of genital warts, vaginal or vulvar cancer, CIN, adenocarcinoma in situ (AIS), or invasive lesions related to HPV 6, 11, 16, or 18. The effectiveness was 100% for preventing HPV-16/18-related high-grade cervical lesions [15]. In a subsequent combined analysis (FUTURE II Study Group, NCT00092534) of four clinical trials (including FUTURE I), 2083 women aged 16–26 years were randomized to receive the 4vHPV vaccine (*n* = 9.087) with a follow-up period of three years. It showed that vaccination was highly effective (efficacy of 99%, CI 93–100%) in preventing HPV-16/18-related precancerous lesions [16].

The 2vHPV has been tested in randomized clinical studies. In a randomized, double-blind, controlled trial conducted on 1113 individuals aged between 15 and 25 years who received three doses of 2vHPV vaccine or placebo, vaccine efficacy at 18 months of follow-up was 91.6% (95% CI 64.5–98.0) against HPV infection and 100% against persistent infection (47.0–100) with HPV-16/18 [17]. At four and a half years of follow-up, there was an efficacy level of 96.9% (95% CI 81.3–99.9) for incident HPV-16/18 infections and 100% (95% CI 42.4–100) for cervical lesions associated with HPV types included in the vaccine [18]. In summary, both 2vHPV and 4vHPV vaccines have shown high efficacy in preventing HPV 16/18 related lesions; the 4vHPV vaccine is also effective against genital warts and other genital lesions [15,16].

In particular, the 4vHPV vaccine demonstrated long-term effectiveness for 10–12 years in a naïve population [17]. Moreover, both vaccines have shown clinical efficacy in women up to 45 years [18,19], and sustained immunogenicity for 2vHPV was elicited in women until 55 years of age [20].

A clinical 9vHPV vaccine study was conducted on 7106 and 7109 women who received the 9vHPV or 4vHPV vaccine, respectively. The 9vHPV vaccine showed about 97% efficacy for high-grade cervical, vulvar, and vaginal disease associated with HPV 31, 33, 45, 52, and 58. It resulted in a non-inferior antibody response to the 4vHPV vaccine for HPV types 6, 11, 16, and 18 [21,22]. In a further study, the analysis of the efficacy of the 9vHPV vaccine was tested by comparing the 9vHPV vaccine arm (7106 women) with the placebo arm of the 4vHPV vaccine efficacy trials (FUTURE I and FUTURE II) [15,16]. A decreased incidence of high-grade cervical lesions (98.2%; 95% CI 93.6–99.7) and cervical surgery (97.8%; 95% CI, 93.4–99.4) related to the nine HPV types was demonstrated [23]. In summary, the 9vHPV vaccine significantly provides additional protection against cervical, vulvar, and vaginal diseases related to targeted HPV genotypes included in the vaccine.

During post-marketing surveillance, the 4vHPV and 2vHPV vaccines have shown to be comparable for safety profiles and demonstrated to be well tolerated. Adverse events usually have a short duration and are non-serious, such as injection-site swelling, fatigue, headache, and myalgia. Fever after injection is reported, but a temperature above 39 °C is rare. In all studies, the rate of systemic severe adverse events is below 0.1% [24,25,26,27,28].

One randomized phase III study on the safety of the 9vHPV vaccine showed a safety profile comparable to that of the 4vHPV vaccine, with only a high incidence of injection-site swelling in the 9vHPV vaccine group [29]. The Global Advisory Committee on Vaccine Safety (GACVS) monitored severe systemic adverse events after introducing HPV vaccines. They included anaphylaxis, syncope, psychogenic illness, multiple sclerosis or other demyelinating diseases, autoimmune diseases, and venous thromboembolism. They were confirmed as not related to vaccination [13,28,30]. HPV vaccination was linked to dysautonomia, chronic fatigue syndrome (CFS), Complex Regional Pain Syndrome (CRPS), and Postural Tachycardia Syndrome (POTS), leading to a vaccination crisis in Japan and Denmark [31,32]. As of now, the American Autonomic Society (AAS) stated that the data might eventually support only a weak temporal association between those events and not a causal relationship [33]. Therefore, the GACVS of the WHO classified these vaccines as ”extremely safe”, even following long-term observations. This has been followed by other national agencies such as the EMA, CDC, the International Federation of Gynecology and Obstetrics (FIGO), and ESGO-EFC [34,35].

## 3. Target Populations

Young girls represent the target population for vaccination programs. The WHO recommends girls aged 9–14 years before becoming sexually active as the primary target group for HPV vaccination. After vaccine introduction, targeting multiple age cohorts between 9 and 18 years led to a higher and faster population impact (herd effect, indirect protection of unvaccinated women, direct protection of boys and men) compared to vaccination of single age cohorts. This approach should also be useful for developing countries because it could guarantee programs are more resilient to any interruptions in vaccine supply. However, a systematic review of the literature showed that too few states have a high vaccination coverage, without a catch-up program to recover further targeted population from vaccinating multiple age cohorts (vs. a single group) [13,36].

Vaccination of secondary target populations, such as older adolescent females or young women or males, is not recommended as a priority. In October 2018, the Food and Drug Administration extended the nonavalent HPV vaccine to men and women aged 27–45 years. It could be proposed that it does not remove resources from the primary objective of vaccination and cervical cancer screening programs [13,37].

The decision to extend vaccination to the male population over the years came from different reasons: the increase in HPV pathology in males (with a burden comparable to that in women in economically developed countries); the proven efficacy of the 4vHPV vaccine versus anogenital warts and anal precancerous lesions (AIN 2–3) [38,39]; the existence of a male subpopulation at higher risk (men having sex with men (MSM), HIV+); social fairness and gender equality rationales, coupled with prevention purposes.

Based on these reasons, since October 2011, the ACIP has recommended routine use of the 4vHPV vaccine in males aged 11 or 12 years, and catch-up vaccination for males aged 13–21 years if unvaccinated. Gender-neutral vaccination has also been recommended in Canada and Australia. In recent years, several other European countries have expanded or are going to expand vaccination to young males, including Germany [40], Italy, Liechtenstein, Norway [41], the United Kingdom [42], Austria, Croatia, Czech Republic and Denmark [43]. In 2017, the Italian government introduced free HPV vaccination for males and females at the age of 12 [44].

It is estimated that by 2015, about 14 million European girls had received full HPV vaccination and 17 million had received at least one dose; with these numbers, 76,000 cases of cervical cancer could be potentially prevented [45]. Considering that 94.3% of vulvar cancers and 87.1% of vaginal cancers are due to HPV genotypes included in the nonavalent vaccine, in this context, the prevention impact is very high [46]. In contrast, oral squamous cell carcinoma is caused in 75% of cases by alcohol and tobacco use, and only 10% of these cancers are due to high-risk HPV genotypes [46]. However, 75–90% of the latter cases are due to HPV genotypes included in the nonavalent vaccine [46].

## 4. Dosing Schedules

The optimal timing for HPV vaccination would be before the sexual debut. Routine HPV vaccination is recommended at 11–12 years. However, it can be initially administered at the age of nine [47].

All the most relevant randomized controlled trials (RCTs) evaluating the effectiveness of three HPV vaccines in preventing high-grade cervical lesions used a three-dose vaccination schedule [48]. More straightforward HPV immunization schedules have been identified as a potential strategy to improve vaccination coverage [49].

The WHO, the Cochrane library, the European Society of Gynecologic Oncology, and the European Federation for Colposcopy recently recommended a two-dose schedule with an adequate distance between the two doses (0, 6–15 months) in young girls aged 9–14 years [17,35,50,51,52,53,54,55]. The WHO position paper reports that patients 15 years and older should receive a 3-dose schedule (0, 1–2, 6 months) [13].

Two doses were found to be non-inferior compared with three doses for all HPV genotypes included in vaccines (bivalent, quadrivalent, and 9-valent vaccines), except for HPV 45 (where non-inferiority was inconclusive), at short-term follow-up (certainty of the evidence GRADE: moderate/high). One-dose regimens are being studied, and to date, they cannot be recommended [55].

In Italy, the vaccine is recommended for all girls and boys during the 12th year of age [56]. The recommended dosing schedule depends on the patient’s age at vaccine initiation and the different HPV vaccine formulation [56]: Table 1.

## 5. Choice of Vaccine

Not all vaccines are available in all regions. In the absence of limitations related to availability and costs, the 9-valent vaccine is recommended. The HPV vaccine can be administered in conjunction with other live and non-live vaccines. Different syringes and injection sites should be used. The best method would be to always apply the same vaccine for all doses. However, if the vaccine used for the previous dose is unknown, any HPV vaccine can be administered to complete the vaccination course.

Long-term follow-up data have shown high antibody titers and clinical efficacy for up to 12 years after vaccination. To date, there is no data to recommend a booster dose [19].

For those women already vaccinated with the bivalent or quadrivalent HPV vaccine, a further full course of three doses of the 9-valent HPV vaccine (2 doses for those younger than 15 years) is possible to achieve complete protection against the additional HPV types [57]. Moreover, the safety and immunogenicity of 9-vHPV were demonstrated in women aged 12–26 years who were previously vaccinated with the 4vHPV vaccine [58].

Although such a strategy is possible, the cost-effectiveness evaluation does not allow for the recommendation of a "revaccination" in population-based programs.

## 6. Post-Treatment HPV Vaccine

After local treatment for high-grade CIN, 5–10% of women may experience high-grade cervical lesion recurrences during follow-up. In these women, the risk of invasive cervical cancer is two to four times higher than in the general population. In contrast, literature data showed a decreased disease recurrence in women treated for CIN who underwent prophylactic vaccines [59]. Observational studies provided indirect evidence of a significant reduction in HPV-related lesions following post-treatment vaccination [60,61,62,63,64]. A prospective Italian study with a 4 vHPV vaccine reported a recurrence rate in 6.4% of unvaccinated women and 1.2% in women vaccinated 30 days after excisional treatment for CIN2+ (*p* = 0.01). There was a reduced risk of recurrent disease in the order of 80% [63]. However, two studies on the 2vHPV vaccine did not confirm the previous findings [65,66]. Despite two studies pointing in different directions, the available evidence [67] and two recently published meta-analyses [68,69] suggest that prophylactic or adjuvant HPV vaccination reduces the risk of recurrent CIN2+. Prospective randomized trials are necessary to confirm these interesting preliminary findings. A prospective multicenter Italian, randomized, double-blind, placebo-controlled, phase III study (“HOPE9 STUDY” HPV vaccine Opportunity Post-surgical Excision ClinicalTrials.gov: NCT03848039) will start in 2020 to investigate the efficacy of presurgical 9-vHPV vaccination in women (18–65 years) treated with LEEP for CIN 2+ and initially invasive cervical cancer (≤ Ia1 FIGO). Moreover, a prospectively designed RCT (NOVEL trial—ClinicalTrials.gov: NCT03979014—started recruitment in 2019) in women treated and then randomized to vaccination or placebo will provide further evidence to assess the cost-effectiveness of post-treatment vaccination.

## 7. Strategy for Implementation

The HPV vaccination represents a paradigmatic example of the under-use of a high-value resource. Although the tests of effectiveness have gradually been consolidated in recent years, and the monitoring of adverse events has shown that HPV vaccines have an adequate safety profile, vaccination coverage in Italy has steadily decreased [70]. In 2017, based on a national survey on adolescents, it was estimated that vaccine coverage among females aged 12 (year of birth: 2005) was 66.6% for at least one dose and only 56.2% for at least two doses [70].

The average vaccination coverage for HPV in girls is fair compared to other European countries, but well below the optimal threshold set by the National Vaccinal Prevention Plan (95%) [68]. In the new Italian National Vaccinal Prevention Plan 2017–2019, free vaccination during the twelfth year of age is expected for males, starting from the 2006 cohort [71].

To increase vaccination coverage, patient reminders, physician-focused interventions (alerts to remind physicians to offer vaccinations), school-based vaccinations programs, and social marketing strategies have proven effective [72].

A systematic review of studies assessing the effectiveness of these interventions showed an improvement in at least one vaccination outcome (higher number of doses) [72].

## 8. Special Populations

The HPV vaccine is safe for HIV or immunocompromised patients [12,73]. The HPV vaccine is also recommended in an at-risk male subpopulation, such as MSM. HPV vaccination should be avoided in pregnant women due to the absence of data in the literature [13]. If a woman becomes pregnant during vaccination, subsequent doses should be postponed until after childbirth [13], but no intervention is needed. Indeed, no increased risk for significant congenital disability [74], spontaneous abortion, stillbirth, or one-year infant mortality was reported following unintended HPV vaccination during pregnancy [75]. On the contrary, breastfeeding is not a contraindication [13]. Travelers and healthcare professionals should follow recommendations like the general population [13].

Special high-risk clinical conditions may also be considered as a new potential target population: patients with inflammatory bowel disease (IBD), patients under biological treatment, transplanted subjects [76], and subfertile men/couples [77,78,79,80,81]. How the issue of HPV vaccination can be addressed in reproductive health to prevent subfertility and/or reproductive failure (embryo quality) in couples that seek pregnancy naturally or through in vitro fertilization is still unsettled. Although meta-analysis suggests that HPV seminal infection can affect male fertility, the exact pathophysiological mechanism remains unclear [82]. A recent study described a new molecular approach to distinguish different HPV genotypes’ sperm infections and to observe the possible effects on semen [79]. Furthermore, the reported positive impact on the reproductive outcome of adjuvant HPV vaccination in infertile couples with HPV semen infection should be further investigated [83,84,85]. If these results are confirmed, they could modify the clinical management of subfertile couples: from HPV screening in the semen of donors or partners to the utility of HPV vaccine in primary prevention of HPV semen infection.

## 9. Conclusions

The introduction of the HPV vaccine, together with HPV screening tests, can potentially minimize malignant and premalignant cervical pathology. Adherence to screening and vaccination programs is critical to achieving this goal. Even though good results have been achieved in industrialized countries, there is still much to do in developing countries. Moreover, the local Italian institutions should plan, organize, and manage the vaccination offer, guaranteeing equity, and universality in access for the most vulnerable population groups. Further studies could provide additional evidence of the role of HPV vaccines after local treatment. The Italian Society of Colposcopy and Cervico-Vaginal Pathology (SICPCV) is committed to supporting vaccination programs for children and adolescents with a catch-up program for young adults. The SICPCV also helps clinical and information initiatives in developing countries to decrease the incidence of cervical pathology.

## Figures and Tables

**Table 1:** Italian HPV vaccine schedules.

HPV Vaccine Formulation	Age (Females)	Doses	Schedule
**Bivalent vaccine**	9–14 *	2 doses	dose 1: 0 mo
			dose 2: 5–7 mo
	over 14	3 doses	dose 1: 0 mo
			dose 2 ^+^: 1 mo
			dose 3 ^+^:6 mo
**Quadrivalent vaccine**	9–13 *	2 doses	dose 1: 0 mo
			dose 2 ^^^: 6 mo
	9–13 *	3 doses	dose 1: 0 mo
			dose 2 ^§^: 2 mo
			dose 3 ^§^: 6 mo
	14 and over	3 doses	dose 1: 0 mo
			dose 2 ^§^: 2 mo
			dose 3 ^§^: 6 mo
**Nonavalent vaccine**	9–14 *	2 doses	dose 1: 0 mo
			dose 2 ^^^: 5–13 mo
	9–14 *	3 doses	dose 1: 0 mo
			dose 2 ^§^: 2 mo
			dose 3 ^§^: 6 mo
	over 14	3 doses	dose 1: 0 mo
			dose 2 ^§^: 2 mo
			dose 3 ^§^: 6 mo

mo: month; * included; ^+^ the second dose should be administered 1 to 2.5 months after the first dose, the third dose 5 to 12 months after the first dose; ^^^ if the second dose is administered five months or earlier after the first dose, a third dose should always be administered; ^§^ the second dose at least one month after the first dose and the third dose at least three months after the second dose; the three doses must be administered within one year.
